# Endogenous conversion of n-6 to n-3 polyunsaturated fatty acids facilitates the repair of cardiotoxin-induced skeletal muscle injury in *fat-1* mice

**DOI:** 10.18632/aging.202655

**Published:** 2021-03-10

**Authors:** Zheng-Gang Wang, Zi-Qing Zhu, Zhi-Yi He, Peng Cheng, Shuang Liang, An-Min Chen, Qing Yang

**Affiliations:** 1 Department of Orthopedics, Tongji Hospital, Tongji Medical College, Huazhong University of Science and Technology, Wuhan 430030, Hubei Province, PR China

**Keywords:** n-3 PUFAs, fat-1 mice, skeletal muscle, injury

## Abstract

In this study, we investigated the beneficial effects of high endogenous levels of n-3 polyunsaturated fatty acids (PUFAs) on skeletal muscle repair and regeneration using a mouse cardiotoxin (CTX, 20 μM/200 μL) -induced gastrocnemius muscle injury model. Transgenic *fat-1* mice expressing the *Caenorhabditis elegans fat-1* gene, encoding n-3 fatty acid desaturase, showed higher n-3 PUFA levels and lower n-6/n-3 PUFA ratios in gastrocnemius muscle tissues. Hematoxylin and eosin and Masson’s trichrome staining of gastrocnemius sections revealed increased muscle fiber size and reduced fibrosis in *fat-1* mice on days 7 and 14 after CTX injections. Gastrocnemius muscle tissues from *fat-1* mice showed reduced inflammatory responses and increased muscle fiber regeneration reflecting enhanced activation of satellite cells on day 3 after cardiotoxin injections. Gastrocnemius muscle tissues from cardiotoxin-treated *fat-1* mice showed reduced levels of pro-apoptotic proteins (Caspase 3 and Bax) and increased levels of anti-apoptotic proteins (Bcl-2 and Survivin). Moreover, eicosapentaenoic acid (EPA) reduced the incidence of apoptosis among cardiotoxin-treated C2C12 mouse myoblasts. These findings demonstrate that higher endogenous n-3 PUFA levels in *fat-1* mice enhances skeletal muscle repair and regeneration following cardiotoxin-induced injury.

## INTRODUCTION

Skeletal muscles are the most dynamic and plastic tissues in the human body, and play an important role in various physiological functions such as motility and metabolism [1]. The loss of muscle mass and strength, especially in the elderly, impairs the ability of the body to respond to stress and chronic disease [2]. Skeletal muscle injury is common during activities of daily living because of strain, trauma, and strenuous exercise, and can lead to muscle atrophy, contracture, and pain [3, 4]. The inflammatory response to muscle injury activates satellite cells and regulates the regeneration of myofibers [3, 5]. The three main phases in the process of muscle healing are (1) initial inflammatory response; (2) regeneration phase that involves activation and proliferation of satellite cells; and (3) remodeling phase that involves maturation of the regenerated fibers [4]. The orderly regeneration of the skeletal muscle is essential for restoring its function after injury and relies significantly on the heterogeneous population of muscle satellite cells [4, 6]. The satellite cells are muscle-resident myogenic stem cells located between the sarcolemma and the basal lamina that undergo proliferation, myogenic differentiation, and fusion into newly formed myofibers in response to muscle injury [6, 7].

The n-3 and n-6 polyunsaturated fatty acids (PUFAs) are essential lipids that are obtained through diet and play a crucial role in several biological processes including metabolism [8]. A high n-6/n-3 PUFA ratio is associated with the pathogenesis of several human diseases [9]. Dietary supplementation of n-3 PUFAs decreases the production of several pro-inflammatory mediators [8, 10] and alleviates inflammatory diseases such as osteoarthritis [11], atopic dermatitis [12], and asthma [13]. As previously reported, inflammatory responses are closely related to muscle injury and repair. Meanwhile, several studies have reported beneficial effects of n-3 PUFAs in disuse-induced skeletal muscle atrophy [14], exercise fatigue [15], and enhancement of muscle strength [16]. However, the role of n-3 PUFAs in skeletal muscle injury has not been explored.

*Fat-1* transgenic mice express the *Caenorhabditis elegans fat-1* gene that encodes n-3 fatty acid desaturase enzyme, which endogenously converts n-6 PUFAs to n-3 PUFAs [17]. The benefits of using *fat-1* transgenic mice include avoiding confounding factors that are associated with dietary supplementation of n-3 PUFAs. Therefore, in this study, we investigated the beneficial effects of high endogenous n-3 PUFA levels on skeletal muscle repair and regeneration after cardiotoxin-induced injury in *fat-1* transgenic mice.

## RESULTS

### *Fat-1* mice express the *fat-1* gene and have high levels of n-3 PUFAs and low n-6/n-3 PUFAs ratio

**Figure 1:**
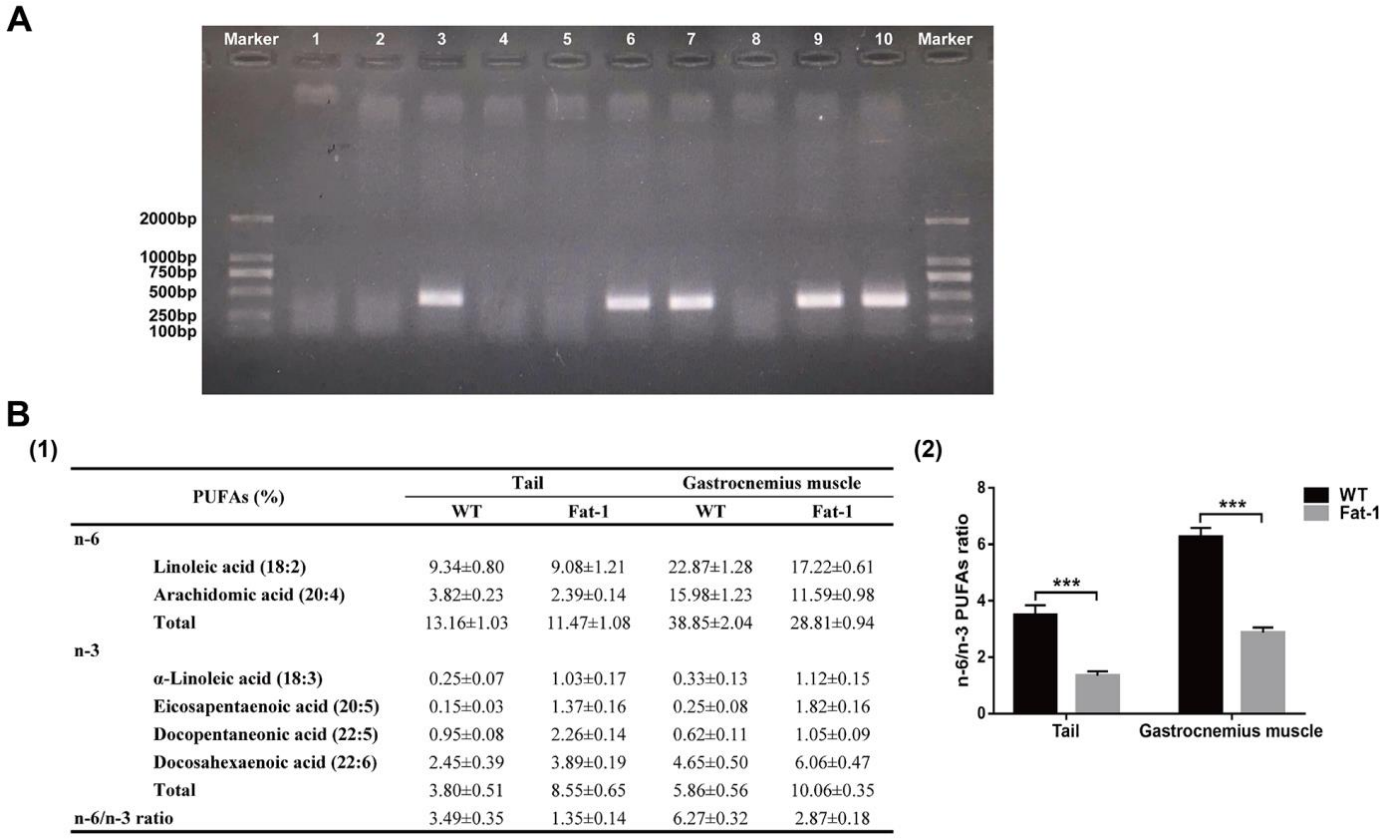
**Fat-1 mice expressed the fat-1 gene and had high levels of n-3 PUFAs and low n-6: n-3 ratio.** (**A**) Genotyping results of *fat-1* gene in *fat-1* and wild-type littermates. (**B**) The lipid composition including n-6 and n-3 PUFA levels and n-6/n-3 PUFA ratio in the tail and gastrocnemius muscle tissues of *fat-1* and wild-type mice. Data were expressed as the percentage of total fatty acids ± SD. ***P < 0.001 vs. WT.

The offspring were identified by genotyping of the 250~500-bp PCR product. There was a representative gel showing samples from WT mice which do not express the *fat-1* gene at lines 1, 2, 4, 5, and 8 compared with samples from *fat-1* mice at lines 3, 6, 7, 9, and 10 (Figure 1A). Furthermore, n-3 PUFA levels were significantly higher and the n-6/n-3 PUFA ratio was significantly lower in the tail and gastrocnemius muscle tissue samples of *fat-1* mice compared to the wild-type mice (Figure 1B).

### High endogenous n-3 PUFA levels attenuates cardiotoxin-induced skeletal muscle injury

**Figure 2:**
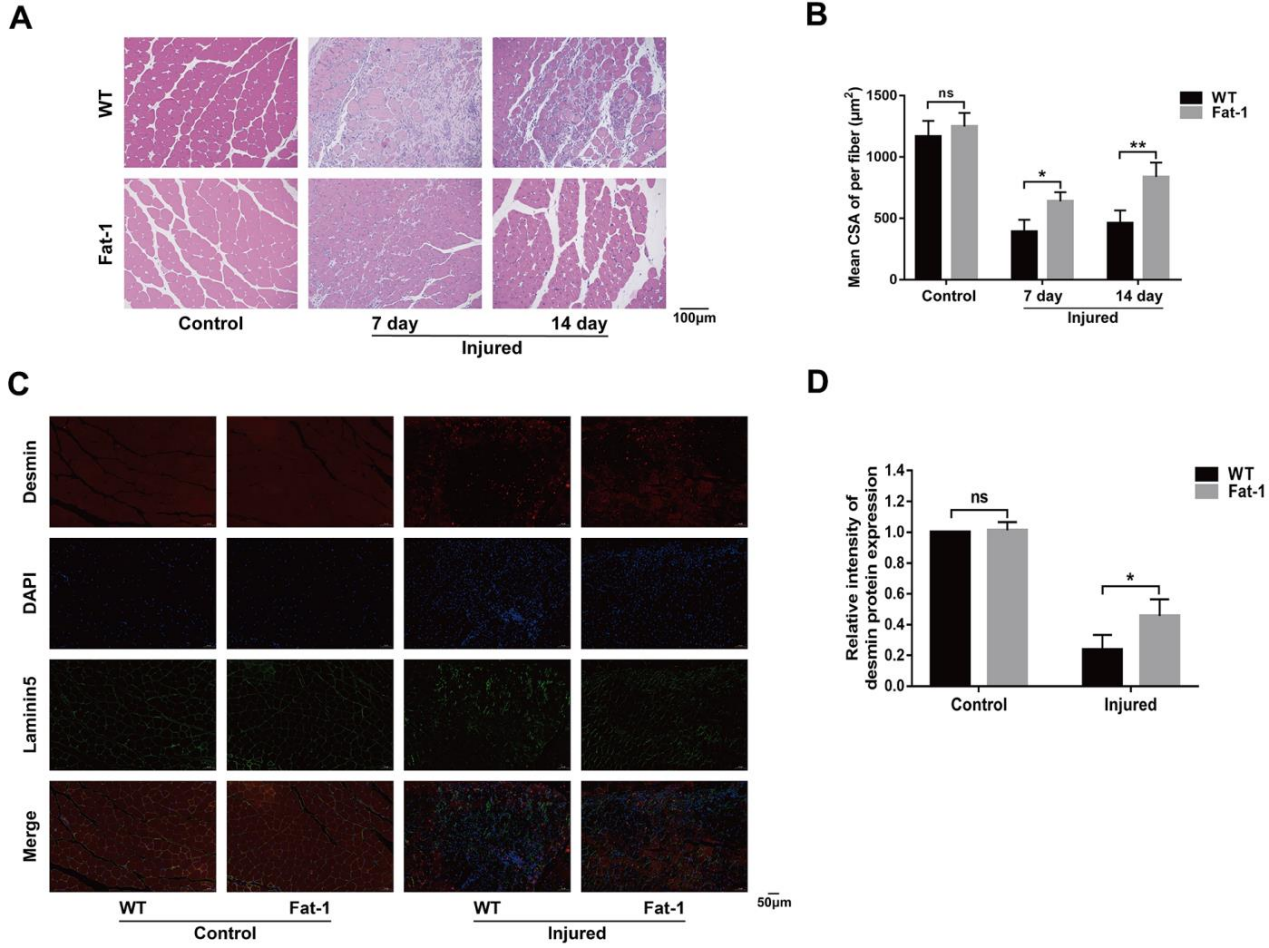
**High endogenous n-3 PUFA levels attenuates cardiotoxin-induced skeletal muscle injury.** (**A**) Representative images show H&E stained gastrocnemius muscle sections of *fat-1* and wild-type mice on days 7 and 14 days after cardiotoxin-induced injury. Scale bar = 100 μm. (**B**) Quantitative data analysis shows the mean cross-sectional muscle fiber area. (**C**, **D**) Representative immunofluorescence images and quantitative data for the intensity of desmin protein expression at 7 days after injury. Scale bar = 50 μm. Data were expressed as the mean ± SD (n = 4-5). NS, non-significant; * *P* < 0.05, ** *P* < 0.01 vs. WT.

**Figure 3:**
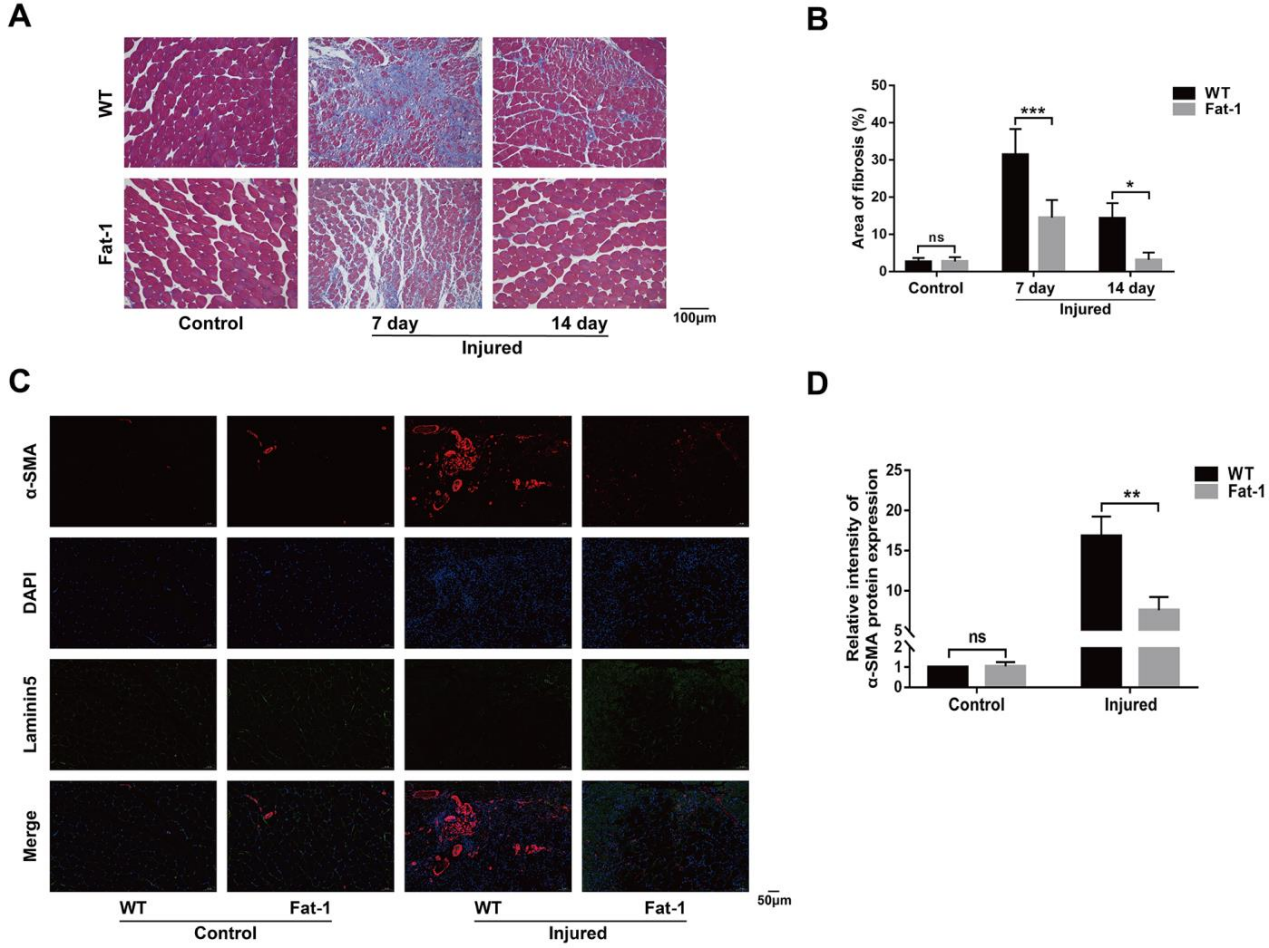
**High endogenous n-3 PUFA levels reduce inflammation response to cardiotoxin-induced muscle injury in fat-1 mice.** (**A**) Representative images show Masson's trichrome staining of the gastrocnemius muscle sections from *fat-1* and wild-type mice on days 7 and 14 days after CTX-induced injury. Scale bar = 100 μm. (**B**) Quantitative data for the fibrosis area. (**C**, **D**) Representative immunofluorescence images and quantitative analysis show α-SMA protein expression in the gastrocnemius muscle tissues from *fat-1* and wild-type mice on day 7 after CTX-induced injury. Scale bar = 50 μm. Data were expressed as the mean ± SD (n = 4-5). NS, non-significant; * *P* < 0.05, ** *P* < 0.01, *** *P* < 0.001 vs. WT.

Next, we evaluated if endogenous levels of n-3 PUFAs alleviate muscle injury by analyzing histological changes in the gastrocnemius muscle tissues of *fat-1* mice compared to the wild-type mice after CTX-induced muscle injury. H&E staining analysis showed that muscle fiber size was better preserved in the fat-1 mice compared to the wild-type mice on days 7 and 14 after injury (Figure 2A, 2B). Next, we performed immunofluorescence labeling using antibodies against desmin, laminin5, and DAPI (Figure 2C). Desmin is an intermediate filament protein that is highly expressed in the immature muscle fibers during regeneration [18]. The intracellular desmin expression was significantly higher in fat-1 mice on day 7 after CTX-induced muscle injury compared to the wild-type mice (Figure 2D). We then quantified the degree of muscle fibrosis using Masson's trichrome staining of gastrocnemius muscle tissue sections of *fat-1* and wild-type mice subjected to CTX-induced muscle injury. The histological analysis showed that muscle fibrosis was significantly lower in the *fat-1* mice compared to the wild-type mice on days 7 and 14 after CTX-induced muscle injury (Figure 3A, 3B). Moreover, immunofluorescence results showed that the levels of α-SAM, a classic myofibroblast marker, were significantly lower in the *fat-1* mice compared to the wild-type mice (Figure 3C, 3D). Taken together, these data demonstrated that *fat-1* gene expression and high endogenous n-3 PUFA levels preserved the structural integrity of the muscle fibers and increased healing in response to CTX-induced muscle injury.

### High endogenous n-3 PUFA levels reduce inflammation response to cardiotoxin-induced muscle injury in *fat-1* mice

**Figure 4:**
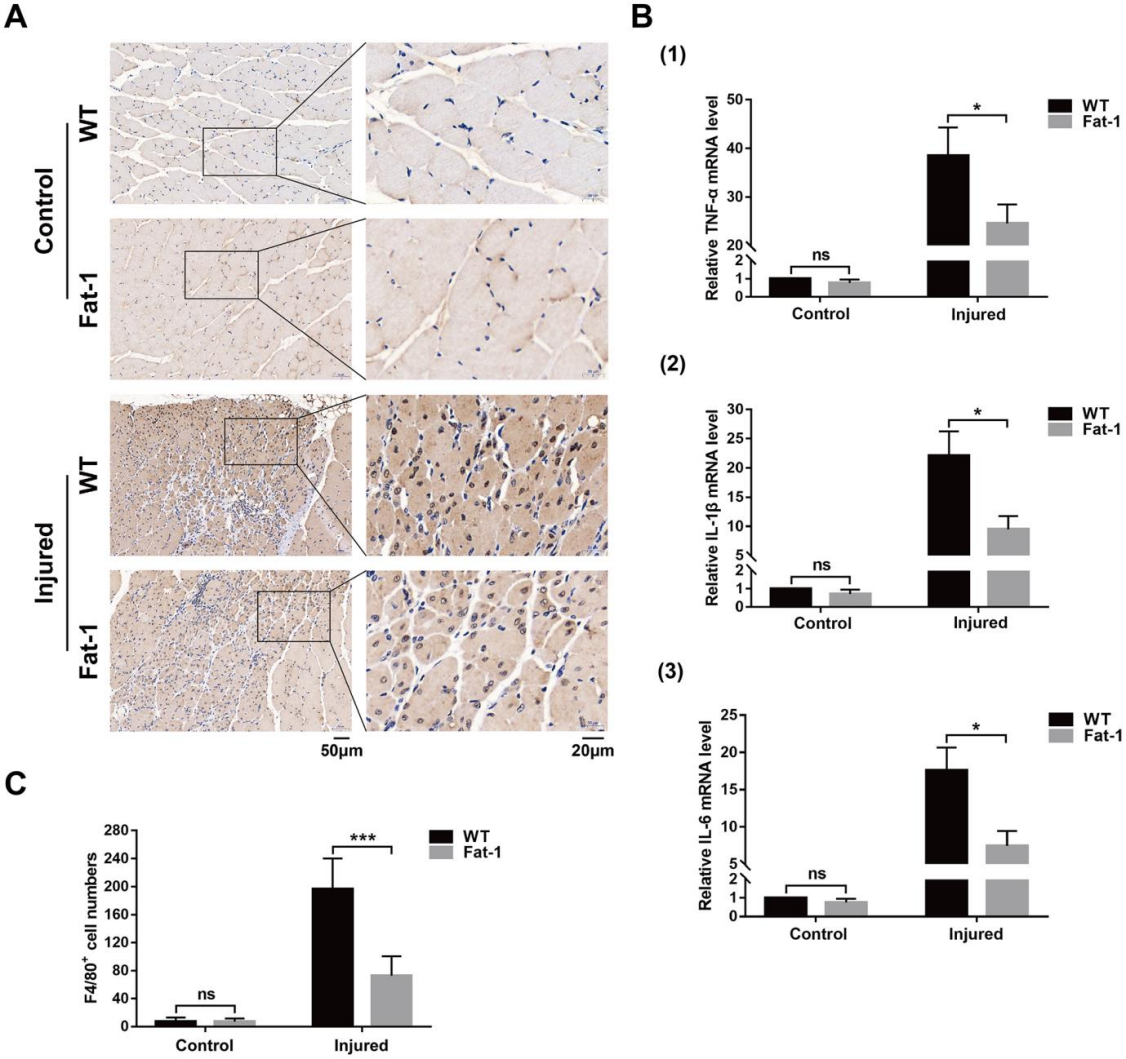
**High endogenous n-3 PUFA levels reduce inflammation response to cardiotoxin-induced muscle injury in fat-1 mice.** (**A**) Representative images show Immunohistochemical staining of the gastrocnemius muscle sections from *fat-1* and wild-type mice with anti-F4/80 antibody on day 3 after CTX-induced muscle injury. Scale bar = 50 μm, 20 μm. (**B**) Quantitative data for F4/80-positive cell numbers. (**C**) Quantitative real-time PCR data shows the relative levels of TNF-α, IL-1β, and IL-6 in the gastrocnemius muscle tissues of *fat-1* and wild-type mice on day 3 after CTX-induced muscle injury. Data were expressed as the mean ± SD (n = 3-4). NS, non-significant; * *P* < 0.05, *** *P* < 0.001 vs. WT.

Inflammation plays a key role at all stages of muscle healing after injury. Moreover, reduced n-6/n-3 ratio in the *fat-1* mice reduces pathology in several models of human inflammatory diseases. We compared the status of inflammatory response in the muscle tissue of *fat-1* and wild-type mice on day 3 after CTX injections. Immunohistochemical analysis with antibody against F4/80, a macrophage marker showed that macrophage infiltration in the gastrocnemius muscle tissue was significantly reduced in *fat-1* mice compared to the wild-type after CTX injection (Figure 4A, 4B). Moreover, qRT-PCR analysis demonstrated that TNF-α, IL-1β, and IL-6 levels were significantly lower in the gastrocnemius muscle of *fat-1* mice after CTX-induced injury compared to the wild-type (Figure 4C). This demonstrated that *fat-1* gene expression suppressed inflammation in response to CTX-induced muscle injury.

### High endogenous n-3 PUFA levels reduce apoptosis in response to cardiotoxin-induced skeletal muscle injury in *fat-1* mice

**Figure 5:**
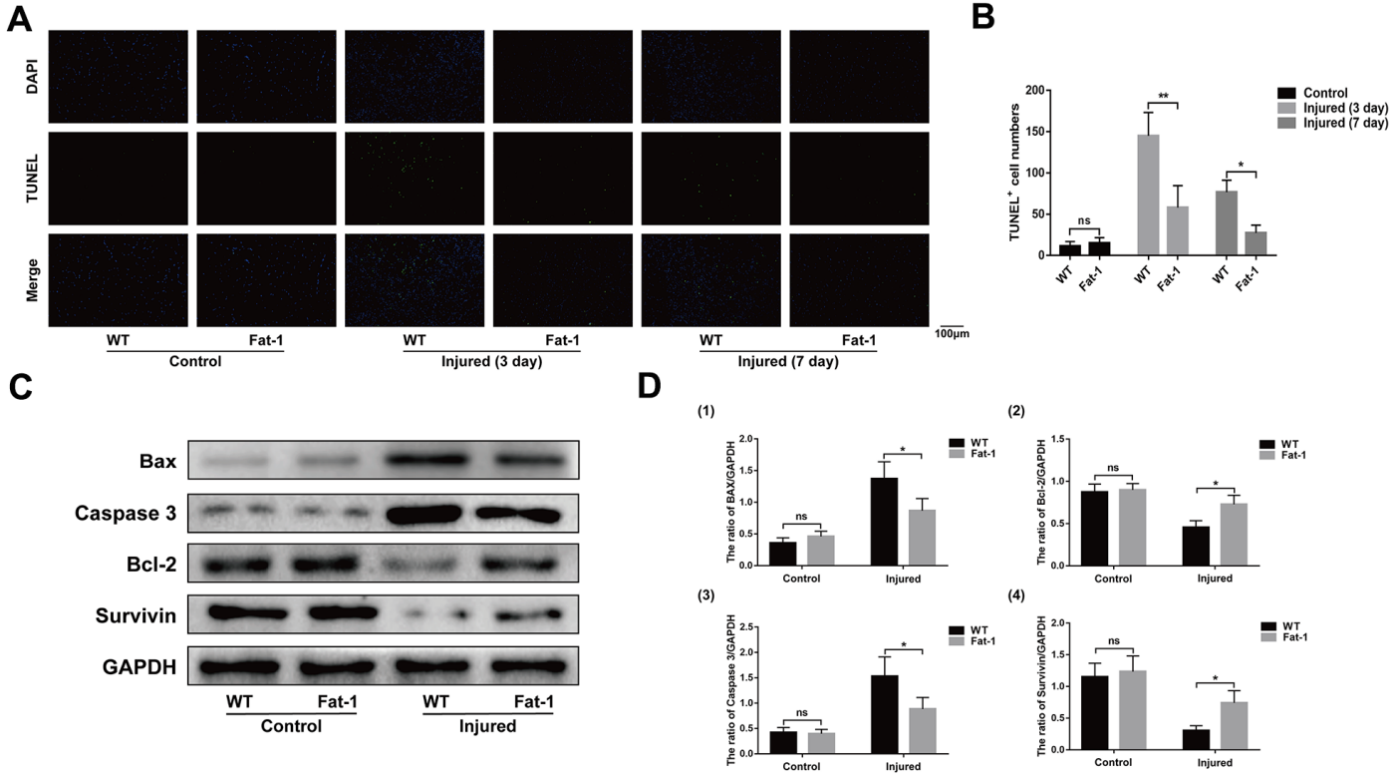
**High endogenous n-3 PUFA levels reduce apoptosis in response to cardiotoxin-induced skeletal muscle injury in fat-1 mice.** (**A**) Representative images show TUNEL staining of the gastrocnemius muscle sections from *fat-1* and wild-type mice on days 3 and 7 after CTX-induced injury. Scale bar = 100 μm. (**B**) Quantitative data for TUNEL-positive cell numbers. (**C**, **D**) Representative western blot images and densitometric analysis show the expression levels of Bax, caspase-3, Bcl-2, and Survivin proteins in the gastrocnemius muscles from *fat-1* and wild-type mice on day 3 after CTX-induced injury. Data were expressed as the mean ± SD (n = 3-4). NS, non-significant; * *P* < 0.05, ** *P* < 0.01 vs. WT.

TUNEL staining analysis showed significantly higher numbers of apoptotic cells in the injured gastrocnemius muscles of the wild-type mice on days 3 and 7 after CTX injection compared to the *fat-1* mice (Figure 5A, 5B). Furthermore, western blot analysis showed that the levels of pro-apoptotic proteins, Bax and Caspase-3, were significantly lower and the levels of anti-apoptotic proteins, Bcl-2 and Survivin, were significantly higher in the gastrocnemius muscles of fat-1 mice on day 3 after CTX-induced injury compared to the wild-type mice (Figure 5C, 5D).

### High endogenous n-3 PUFA levels increase muscle regenerative capacity in response to cardiotoxin-induced skeletal muscle injury in *fat-1* mice

**Figure 6:**
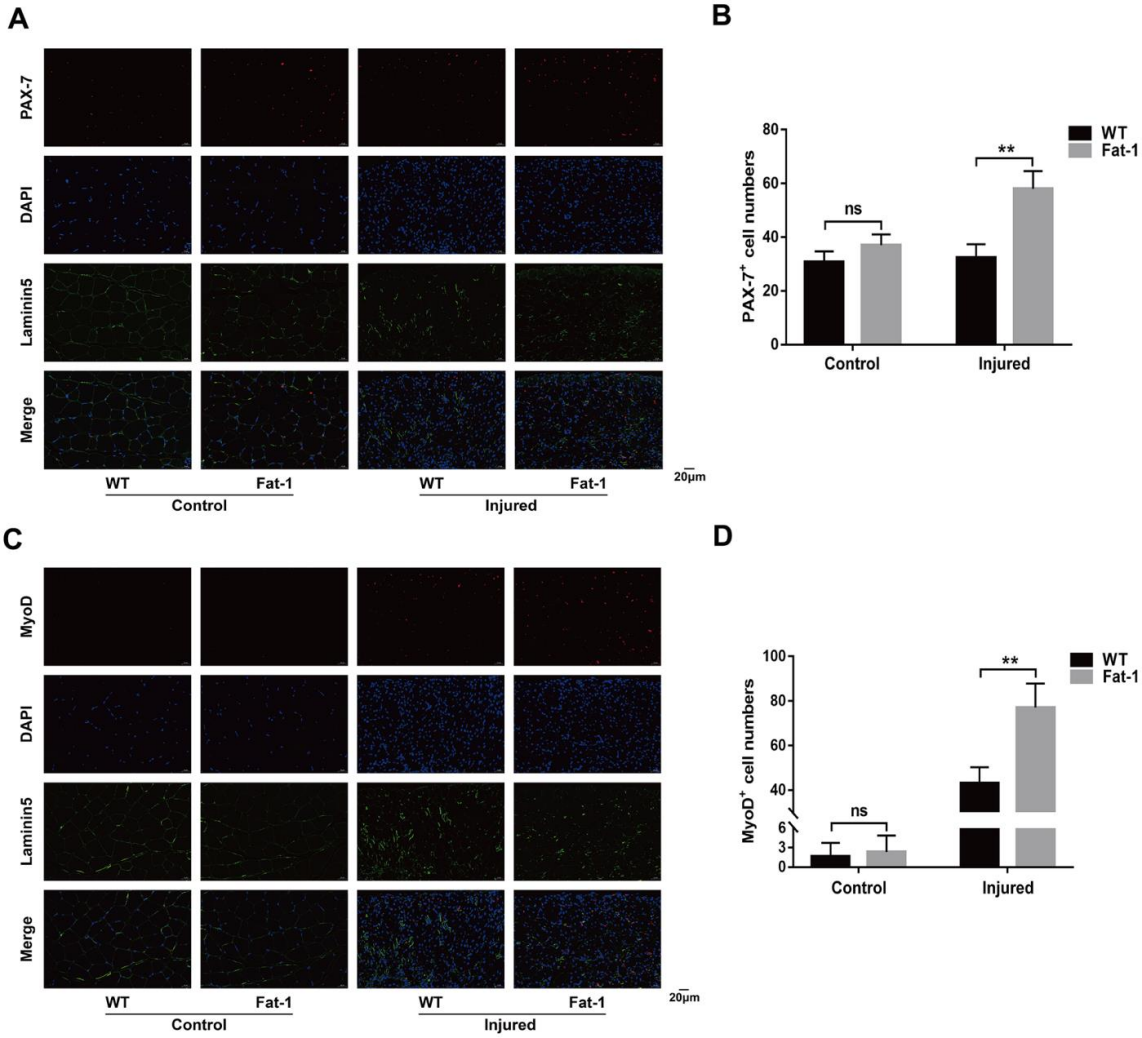
**High endogenous n-3 PUFA levels increase muscle regenerative capacity in response to cardiotoxin-induced skeletal muscle injury in fat-1 mice.** Representative immunofluorescence images and quantitative analysis shows the expression levels of (**A**, **B**) PAX-7 and (**C**, **D**) MyoD proteins in the gastrocnemius muscle tissues from Fat-1 and wild-type mice on day 3 after CTX-induced muscle injury. Scale bar = 20 μm. Data were expressed as the mean ± SD (n = 4-5). NS, non-significant; ** *P* < 0.01 vs. WT.

Next, we tested if higher endogenous levels of n-3 PUFAs promote muscle regeneration after injury by analyzing the expression of PAX-7 and MyoD. PAX-7 is a specific marker of satellite cells and MyoD is a marker for satellite cell activation. Immunofluorescence results showed significantly higher numbers of PAX-7-postitive (Figure 6A, 6B) and MyoD-positive cells (Figure 6C, 6D) in the gastrocnemius muscle sections of *fat-1* mice compared to the wild-type mice on day 3 after CTX-induced muscle injury. These results showed significantly higher rate of satellite cell activation in the gastrocnemius muscle tissues of *fat-1* mice compared to the wild-type mice on day 3 after CTX-induced muscle injury.

### EPA prevents cardiotoxin-induced C2C12 cell apoptosis

**Figure 7:**
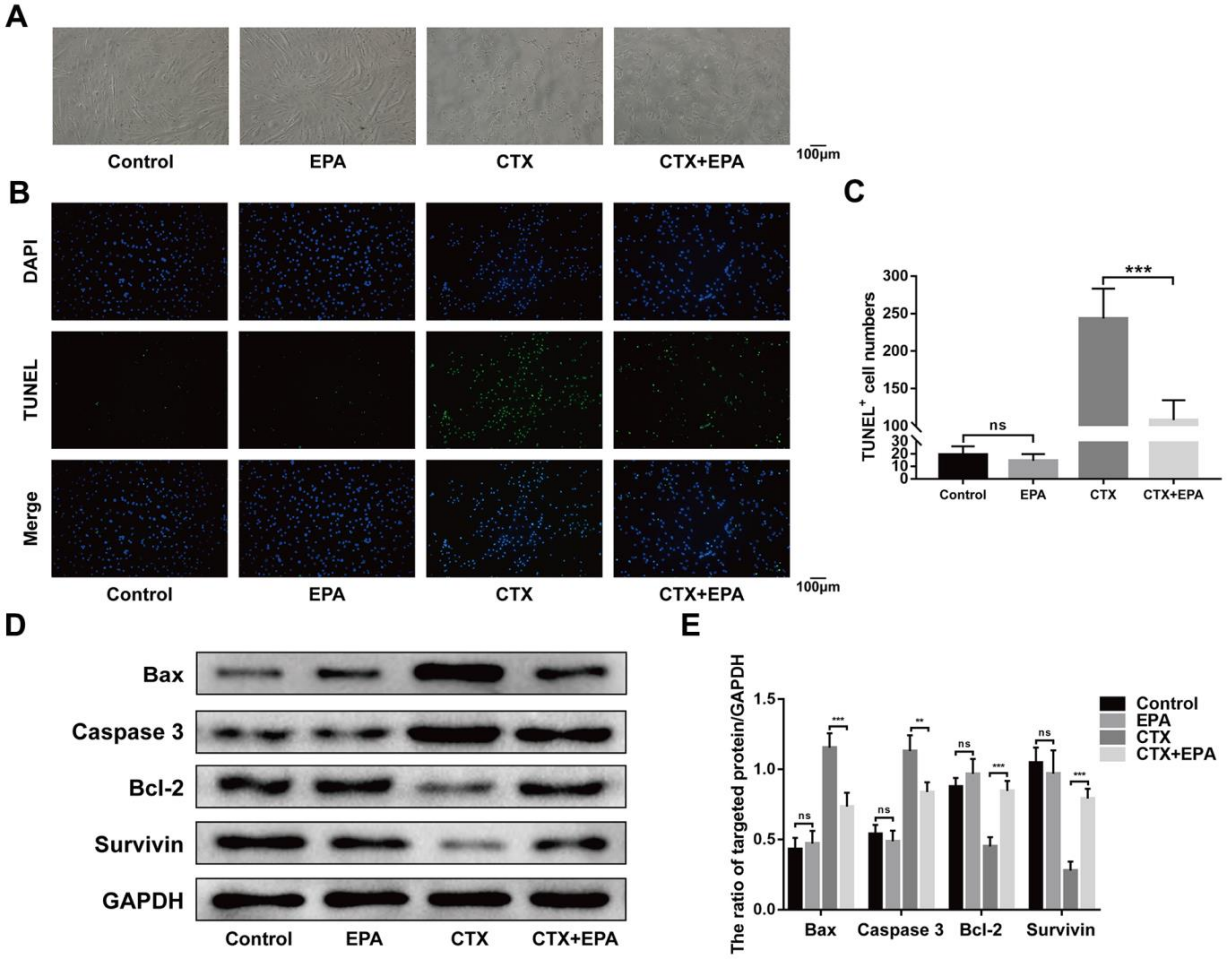
**EPA prevents cardiotoxin-induced C2C12 cell apoptosis.** (**A**) Representative images of morphological change of the differentiated C2C12 cells cultured in the presence and absence of cardiotoxin (0.5 μM) alone and in combination with EPA (50 μM) for 24 h. Scale bar = 100 μm. (**B**, **C**) Representative images of TUNEL staining and quantitative data for TUNEL-positive cell numbers. Scale bar = 100 μm. (**D**, **E**) Representative western blot images and densitometry analysis shows the levels of Bax, caspase 3, Bcl-2, and Survivin in the lysates of the experimental groups. Data were expressed as the mean ± SD (n = 3). NS, non-significant; ** *P* < 0.01, *** *P* < 0.001 vs. Control/CTX+EPA.

EPA is one of the most common long chain n-3 PUFA. Therefore, we investigated if EPA reduced *in vitro* apoptosis in cardiotoxin-induced C2C12 cells. The morphological changes in differentiated C2C12 cells treated with 0.5 μM cardiotoxin with or without 50 μM EPA for 24 h are shown in Figure 7A. TUNEL-staining analysis showed that EPA significantly reduced apoptosis of CTX-induced C2C12 cells compared to the controls (Figure 7B, 7C). Western blot analysis showed that EPA treatment reduced the levels of BAX and Caspase-3 proteins and increased the levels of Bcl-2 and Survivin proteins in cardiotoxin-induced C2C12 cells compared to the corresponding controls (Figure 7D, 7E). Overall, these results showed that EPA suppressed apoptosis of CTX-induced C2C12 cells, thereby confirming the results observed in CTX-induced *fat-1* mice.

## DISCUSSION

Previous studies demonstrated that supplementation of PUFAs in daily diet promotes skeletal muscle function, metabolism, and repair [16, 19–21]. However, the relationship between endogenous PUFAs and the ratio of n-6/n-3 PUFAs with skeletal muscle function and repair is not clear. Marzuca-Nassr et al. reported that balanced diet-fed *fat-1* mice showed reduced soleus muscle atrophy in response to a two-week hind-limb suspension as a model of skeletal muscle atrophy [14]. In this study, we demonstrate that endogenous n-3 PUFAs and decreased n-6/n-3 PUFA ratio in *fat-1* mice significantly protects against cardiotoxin-induced skeletal muscle injury.

EPA is a substrate for cyclooxygenases and lipoxygenases and induces synthesis of anti-inflammatory compounds such as prostaglandins and leukotrienes by directly competing with arachidonic acid (AA); in combination with docosahexaenoic acid (DHA), EPA promotes production of pro-inflammatory lipid mediators [22–24]. EPA and DHA are also agonists of peroxisome proliferator-activated receptors (PPAR), which are transcription factors, and inhibit activation of NF-κB signaling pathway [25, 26].

Gas chromatography analysis showed that the levels of n-3 PUFAs such as EPA were elevated and the n-6/n-3 PUFA ratio was significantly reduced in the tail and gastrocnemius muscle tissues of *fat-1* mice. A previous study showed that EPA improved the regenerative capacity of mouse skeletal muscle cells [27]. Our study also demonstrated that EPA suppressed apoptosis of cardiotoxin-induced C2C12 cells.

Our study also showed that interstitial fibrosis and α-SMA protein expression was reduced in the gastrocnemius muscle tissues of *fat-1* mice compared to the wild-type mice. Previous studies showed that supplementation of n-3 PUFAs suppressed TGFβ1-induced pro-fibrogenic gene expression in the liver tissues [28] and inhibited interstitial fibrosis in transplanted kidneys [29]. Endo et al reported that fibroblast activation in response to pressure overload was significantly lower in the *fat-1* mice compared to the wild-type mice [30].

The anti-inflammatory mechanisms of n-3 PUFAs are complex and include changes in activation of several transcription factors and subsequent gene expression alterations and inhibition of the synthesis of pro-inflammatory eicosanoids from arachidonic acid [31]. Our study showed significantly reduced macrophage infiltration and gene expression of pro-inflammatory factors in the CTX-induced *fat-1* mice. However, a previous study reported significantly higher inflammatory response in response to a low n-6/n-3 PUFA ratio in the daily diet [32]. Conversely, several studies have reported that supplementation of n-3 PUFAs decreased the levels of pro-inflammatory factors [33, 34]. These contradictory results may due to differences in study design and supplementation. In our study, we used *fat-1* mice to avoid the effects of confounding factors such as animal strains, oxidation of PUFAs in the food, duration of feeding, and contamination of n-3 PUFA supplements with trace elements.

The n-3 PUFAs alter the function of membrane signaling proteins directly or indirectly, thereby modulating the activation of transcription factors and gene expression patterns [22, 35]. In our study, endogenous n-3 PUFAs upregulated the expression levels of anti-apoptotic proteins (Bcl-2 and Survivin) and downregulated the expression levels of pro-apoptotic proteins (caspase 3 and Bax). These results are consistent with previously reported findings in other types of cells [36, 37]. We also found that endogenous n-3 PUFAs influenced the expression levels of Pax-7 and MyoD, thereby promoting muscle regeneration by enhancing the activation and differentiation of satellite cells.

Recently, several experimental studies and clinical trials have documented the beneficial actions of n-3 PUFAs in neuropsychiatric disorders, cardiovascular diseases, obesity, cancers, and diseases related to the musculoskeletal system [38–41]. In elderly individuals, supplementation of n-3 PUFAs improved muscle function and quality, thereby increasing walking speed [42, 43]. Moreover, n-3 PUFA supplementation showed beneficial effects by preventing soft-tissue injuries caused by exercise as well as improving their healing times [23, 44]. A randomized controlled trial showed that n-3 PUFA supplementation decreased disability and pain in individuals suffering from rotator cuff-related shoulder pain [45].

This study has several limitations. Firstly, we did not evaluate the beneficial effects of endogenous n-3 PUFAs on skeletal muscle function. Secondly, the underlying molecular mechanisms related to the protective effects of endogenous n-3 PUFAs in *fat-1* mice against skeletal muscle injury need to be further investigated.

In conclusion, our study demonstrates that endogenous conversion of n-6 to n-3 PUFAs facilitates the repair of cardiotoxin-induced skeletal muscle injury by suppressing inflammation, inhibiting cellular apoptosis, and promoting muscle regeneration in *fat-1* mice. Our study demonstrates potential benefits of clinically treating skeletal muscle injuries with n-3 PUFA supplements.

## MATERIALS AND METHODS

### Gastrocnemius muscle injury mouse model

The heterozygous *fat-1* transgenic mice were obtained from Nanjing Medical University, housed in a temperature-controlled room at a constant temperature of 22° C ± 2° C under a 12h/12h light/dark cycle, and provided with food and water ad libitum. All animal experiments were approved and supervised by the Experimental Animal Ethics Committee of Tongji Hospital, Tongji Medical College, Huazhong University of Science and Technology.

The cardiotoxin-induced gastrocnemius muscle injury model in 3-week-old *fat-1* mice and their wild-type littermates was established as described previously [46]. The mice were killed by cervical dislocation on days 3, 7, and 14 after CTX injections. The gastrocnemius muscles were harvested for further analysis.

### Cell culture

C2C12 mouse myoblast cell line was purchased from the Cell Resource Center, Shanghai Academy of Life Sciences, Chinese Academy of Sciences (Shanghai, China). The cells were cultured in Dulbecco's modified Eagle's medium (DMEM) containing 10% fetal bovine serum (Gibco, Grand Island, NY, USA) and 1% Pencillin/Streptomycin antibiotics in a humidified incubator maintained at 37° C and 5% CO2. When the cells became 90% confluent, they were transferred into a differentiation medium containing 2% horse serum (Solarbio, Beijing, China). The differentiated cells were maintained in DMEM without serum for 6 h. Then, they were pre-treated with different concentrations of EPA, cultured with or without CTX, and then analyzed further for various parameters.

### Genotyping

For genotyping, we obtained 2 mm tail from 2-week old offsprings, extracted total DNA and performed PCR using the Mouse tissue Direct PCR kit (KG205, Tiangen, Beijing, China) according to manufacturer’s instructions. The PCR primers for the *fat-1* gene were as follows: forward, 5’-GGACCTGGTGAAGAGCATCCG-3’; reverse, 5’-GCCGTCGCAGAAGCCAAAC-3’. The PCR products were resolved on a 2% agarose gel with ethidium bromide and the bands for the *fat-1* gene were imaged using the Bio-Rad Gel Doc system.

### Gas chromatography

The murine tail and gastrocnemius muscle tissues were homogenized in a solvent mixture of chloroform: methanol in a 2:1 ratio for extracting total fat. Then, the fatty acids were methylated with 0.25N Sodium methoxide in methanol for 20 minutes at 70° C to obtain fatty acid methylesters (FAMEs). The FAMEs were then extracted by hexane as previously described [47] and analyzed using a gas chromatograph equipped with a capillary column and a flame ionization detector. The head pressure of the carrier gas (helium) was 300 kPa; the temperatures of the injector and detector were 230° C and 260° C, respectively. The initial temperature of the column oven was 170° C for 2 minutes and was sequentially increased at the rate of 1.5° C/min to 220° C. The peaks obtained from the *fat-1* and wild-type murine tails and gastrocnemius muscle tissue samples were compared with fatty acid standards for identification. The percentage composition of each PUFA was evaluated by calculating the integrated area of every resolved peak.

### Histological staining

We cut 5 μm thick cross sections of paraffin-embedded gastrocnemius muscle samples. The sections were stained with hematoxylin and eosin (H&E) or Masson’s trichrome staining, observed and photographed under an optical microscope, and analyzed with the Image J software.

### Immunohistochemistry

The de-paraffinized and rehydrated gastrocnemius muscle sections were blocked in 5% bovine serum albumin (BSA) with 0.1% Triton X-100 for 1 h and incubated with primary antibody against F4/80 (ab111101, Abcam, Cambridge, MA, USA) at 4° C overnight. Then, the sections were incubated with the secondary antibody (Boster, Wuhan, Hubei, China) at room temperature for 1 h. After color development, the sections were observed under a light microscope and the proportions of positively stained cells were analyzed.

### Immunofluorescence

The de-paraffinized and rehydrated sections of gastrocnemius muscle were blocked in 5% BSA with 0.1% Triton X-100 for 1 h and incubated with primary antibodies against Laminin-5 (ab11575, Abcam), Desmin (sc-23879, Santa Cruz Biotechnology, CA, USA), Pax-7 (sc-81648, Santa Cruz Biotechnology), Myod (sc-32758, Santa Cruz Biotechnology), and α-SMA (ab5694, Abcam) at 4° C overnight. Then, after washing three times with PBS, the sections were treated with secondary antibody (Boster) for 1 hour in the darkness. Finally, the sections were washed thrice with PBS, stained with DAPI (Boster) for 10 minutes, and photographed using a fluorescence microscope.

### TUNEL staining

Apoptosis in gastrocnemius muscle tissue samples and C2C12 cells were determined using the TUNEL apoptosis detection kit according to the manufacturer’s instructions. The sections and cells were incubated with DAPI at 37° C to stain the nuclei. The images were captured with a fluorescence microscope and the TUNEL-positive cells were analyzed using the Image J software.

### Western blotting

Total protein lysates were prepared from C2C12 cells and gastrocnemius muscle tissues. Protein concentrations were measured with a BCA assay kit (Boster). Then, equal amounts of protein lysates were separated on 10% SDS-polyacrylamide gels and transferred onto poly-vinylidene fluoride membranes (Millipore, Billerica, MA, USA). The membranes were subsequently blocked with 5% BSA for 1 h and incubated with primary antibodies against Bcl-2 (ab59348; Abcam) and Survivin (ab469; Abcam) Caspase 3 (19677-1-ap; Proteintech, Wuhan, Hubei, China), BAX (5099-2-lg; Proteintech), and GAPDH (60004-1-Ig; Proteintech) at 4° C overnight. This was followed by incubation with HRP-conjugated secondary antibodies (Boster) for 1 h. The blots were developed using Pierce ECL Western Blotting Substrate and visualized using the ChemiDoc XRS System (Bio-Rad Laboratories, Hercules, CA, USA).

### Quantitative real-time polymerase chain reaction

We extracted total RNA from muscle tissues using the Total RNA extraction kit (Toyobo, Osaka, Japan) and quantified it using NanoDrop. Samples of total RNA were reverse transcribed into cDNA, which were then subjected to qPCR using the Bio-Rad system with specific primers. The qPCR primers used in this study were as follows: IL-6 (Forward), 5’-CTGCAAGAGACTTCCATCCAG-3’; IL-6 (Reverse), 5’-AGTGGTATAGACAGGTCTGTTGG-3’; IL-1β (Forward), 5’-GAAATGCCACCTTTTGACAGTG-3’; IL-1β (Reverse), 5’- TGGATGCTCTCATCAGGACAG-3’; TNF-α (Forward), 5’-CAGGCGGTGCCTATGTCTC-3’; TNF-α (Reverse), 5’-CGATCACCCCGAAGTTCAGTAG-3’.

### Statistical analysis

All data are expressed as means± standard deviation (SD). The data between two groups were analyzed by the Student’s t-test. The data between multiple groups were analyzed using One-way analysis of variance (ANOVA). P <0.05 was considered statistically significant. Statistical analysis was performed using the SPSS software for Windows 17.0 (SPSS Inc., Chicago, IL, USA).
